# Enhancing biostatistics education for medical students in Poland: factors influencing perception and educational recommendations

**DOI:** 10.1186/s12909-024-05389-z

**Published:** 2024-04-22

**Authors:** Michal Ordak

**Affiliations:** Department of Pharmacotherapy and Pharmaceutical Care, Faculty of Pharmacy, Medical University of Warsaw, 1 Banacha Street, 02-097 Warsaw, Poland

**Keywords:** Biostatistics, Education

## Abstract

**Background:**

A number of recommendations for the teaching of biostatistics have been published to date, however, student opinion on them has not yet been studied. For this reason, the aim of the manuscript was to find out the opinions of medical students at universities in Poland on two forms of teaching biostatistics, namely traditional and practical, as well as to indicate, on the basis of the results obtained, the related educational recommendations.

**Methods:**

The study involved a group of 527 students studying at seven medical faculties in Poland, who were asked to imagine two different courses. The traditional form of teaching biostatistics was based on the standard teaching scheme of running a test from memory in a statistical package, while the practical one involved reading an article in which a particular test was applied and then applying it based on the instruction provided. Other aspects related to the teaching of the subject were assessed.

**Results:**

According to the students of each course, the practical form of teaching biostatistics reduces the stress level associated with teaching and the student exam (*p* < 0.001), as well as contributing to an increased level of elevated knowledge (*p* < 0.001), while the degree of satisfaction after passing the exam is higher (*p* < 0.001). A greater proportion of students (*p* < 0.001) believe that credit for the course could be given by doing a statistical review of an article or conducting a survey, followed by the tests learned in class. More than 95% also said that the delivery of the courses should be based on the field of study they were taking, during which time they would also like to have the opportunity to take part in optional activities and hear lectures from experts.

**Conclusion:**

It is recommended that more emphasis be placed on practical teaching the subject of biostatistics.

**Supplementary Information:**

The online version contains supplementary material available at 10.1186/s12909-024-05389-z.

## Introduction

In recent years, there has unfortunately been an observed decline in the quality of research results published in articles, attributed to various types of erroneous statistical analyses. Among other things, there is insufficient statistical reporting [1]. Another factor contributing to this challenge is the editors’ difficulty in identifying suitable statistical reviewers. In response, published guidelines, which are my recommendations, strive to alleviate this issue [2]. However, data from PLOS ONE suggest that incorporating statistical reviewers has a beneficial impact on enhancing the quality of manuscripts [3]. There is a growing concern that most of the currently published research findings are false [4]. A greater proportion of scientists recognise that education on biostatistics is important but not sufficient [5]. In order to reduce the problem of the authors’ misguided statistical analysis, the main focus should also be on changing the way the subject of biostatistics is taught. In 2016, PLOS BIOLOGY published a highly pertinent and practical article. The authors aimed to pinpoint opportunities for enhancing the teaching of biostatistics in basic science. The recommendations highlighted are associated with tailoring teaching methods to students’ research fields and fostering biostatistics education through the creation of tools and strategies [6]. The limited understanding of the statistical analyses conducted points to the need for improved teaching in this area [7]. Recommendations published in Nature Medicine indicate that reproducibility of studies shows a relationship with appropriate statistical training [8]. According to further data published in PLOS ONE, students report a neutral perception of the value of biostatistics [9]. Statistics anxiety is frequently viewed as one of the most potent negative influences on performance in statistics courses [10, 11]. Statistical concepts are frequently depicted as challenging, anxiety-inducing, and crucial for the typical medical student [12, 13]. Students very often approach biostatistics with apprehension and treat the subject as a requirement. For this reason, students should be encouraged to bring in their own research papers so that a statistical analysis of their findings can then be carried out in a practical way. One recommendation is to provide practical examples so that students can become even more interested in biostatistics [14]. In a recent study, researchers divided a cohort of 96 students in a recent medical course into two groups: one group attended traditional didactic lectures, while the other engaged in problem-based teaching focused on specific topics in biostatistics. The latter approach demonstrated greater effectiveness [15]. In the 2015 article published in BMC Medical Education, a study on the assessment of statistical needs revealed that the obtained results indicated that 73% of respondents prefer short courses with “hands-on” practice [16]. Despite a number of published recommendations related to biostatistics education, there is a lack of articles in the literature that include students’ opinions on how to teach the subject in practice. Experience to date has shown that most of the teaching done focuses on running a series of test procedures in various statistical packages, rather than on practically exploring the beauty of biostatistics. There is an urgent need for research into the impact of different biostatistics teaching techniques on the level of knowledge gained from the course and the overall satisfaction of students. For this reason, the aim of this manuscript was to compare students’ views on the teaching of biostatistics in two schemes. One was mainly focused on performing a statistical test in the package, while the other was based on recommendations published in PLOS BIOLOGY [6].

## Methods

### Study group

Students from nine medical universities in Poland in their final year of study took part in the study. The choice of these universities was linked, among other things, to getting as many students as possible willing to complete the survey, including courses with a small number of students. In other words, the aim of the research conducted was to collect a minimum of 50 people studying the fields of study indicated below. In order to increase the chances of obtaining such a figure, the survey was conducted among students from nine medical universities in Poland. These were students for whom graduation was linked to the writing of a thesis and thus the need for them to carry out a statistical analysis of the data collected. Students of pharmacy, medical analytics, physiotherapy, dietetics, emergency medicine, midwifery and public health were included in the study. First, contact was made with the supervisor of the specific course and year of study. This supervisor then forwarded the contact to the year head, who then forwarded the created survey to the students of his or her year by email. Such students who had already completed a statistics course were included in the analysis. The selection of specific yearbooks was based on course syllabuses posted on the website, through which it was determined whether students had completed the subject. Participation in the survey was voluntary. Each person completing the survey was made aware in advance of the purpose of the analyses being carried out and informed that the data collection was for the purpose of this research.

### Survey

The study involved students completing a survey created by the author of the manuscript. The questions included in the survey were based on the author’s extensive experience in conducting statistical consultations with groups of students in medical disciplines in Poland, as well as on references specified in the article regarding the justification for practical teaching of biostatistics. Data presented at the International Statistical Congress in Malaysia revealed that 95% of medical students believe insufficient knowledge related to conducting statistical analyses results from four factors: the excessively fast pace of classes, the lack of practical teaching in the subject, long breaks between classes, and the requirement to write master’s theses. The perception is further exacerbated by increased exam stress. This finding prompted the inclusion of these variables in the survey to comprehensively understand students’ opinions on the matter [17]. The initial data analysed included gender, age, place of residence, and field of study. The next stage of the research conducted was based on the same questions, but concerning two different forms of classroom management. The first concerned the traditional form of teaching that takes place in medical studies. It involves the instructor presenting the various procedures involved in running a particular statistical test in the package, followed by the student performing the same task. The second practical part involved, at the outset, an analysis of a selected article in which the authors applied a specific statistical test. Later in the course, the students had in front of them a step-by-step guide on how to carry out the statistical test in question. Thus, they were able to perform the specific task presented to them on its basis and then interpret the findings according to the recommendations they had before them. For each of the two delivery options, students’ opinions on the severity of stress related to the delivery of the class, the exam ahead, were assessed. Other parameters analysed included the degree of satisfaction with the classes and the opinion as to whether practical knowledge could be gained from them. The last part of the survey included questions related to the requirement for staff to attend training courses in biostatistics, but whether classes taken during their studies are sufficient in this respect. The next questions concerned the possible form in which the class could be completed, i.e. analysing the article for statistical validity, or performing a survey and then analysing the results using the statistical test discussed in class. The final questions covered students’ opinions on whether it should be possible to take optional classes in biostatistics, to listen to lectures given by statisticians and whether classes should be based on the field of study being taken. A student-filled survey is included in the first Appendix (Appendix 1).

### Statistical analysis

In order to check whether there were statistically significant differences in the entire group of students surveyed, as well as by their field of study, the Wilcoxon test was used. This choice was made because the normality of the distribution tested was disturbed using the Shapiro-Wilk test, as well as because of the unevenness of the students in the different subjects. The relationship between the nominal variables was analysed using the chi-square test. Descriptive statistics used included mean, standard deviation, median, first and third quartiles. Statistical analysis was performed using the IBM SPSS Statistics v. 25 (IBM Corp., Armonk, NY, USA). A p value < 0.05 was considered statistically significant.

## Results

### Study group

The study involved 527 students from seven medical faculties at nine medical universities in Poland. The following table provides the basic sociodemographic data of the students. The field of study shows a statistically significant relationship with the gender of the subjects, χ^2^(6) = 45.55; *p* < 0.001. The largest number of students were female, while of the male students, the largest number were studying physiotherapy and public health. Place of residence does not show a statistically significant relationship with the field of study, χ^2^(6) = 1.39; *p* = 0.97. Most people were from the city (Table 1). The median age in the medical analyst, pharmacy and paramedic student groups was 24 years, while the median age for the other student groups was 23 years.

**Table 1:** Gender and place of residence of the study group of students

Study subject	Sex				Place of residence				n
	Female		Male		Village		City		
	n	%	n	%	n	%	n	%	
Medical analytics	65	93	5	7	7	10	63	90	70
Dietetics	69	82	15	18	10	12	74	88	84
Pharmacy	95	86	16	14	13	12	98	88	111
Physiotherapy	43	62	26	38	7	10	62	90	69
Midwifery	56	100	0	0	4	7	52	93	56
Public health	57	72	22	28	10	13	69	87	79
Emergency medicine	40	69	18	31	7	12	51	88	58

### Students’ views on the practicalities of teaching biostatistics

As a first step, it was checked whether there were statistically significant differences between the two forms of biostatistics teaching in terms of the variables analysed in each course and in the whole group of people. Given the deviations from the assumption of normality in the distribution of the analyzed variables and the unequal nature of the groups, differences for each direction were tested using the Wilcoxon test. The analysis carried out showed that for each parameter analysed in the table below (Table 2), there were statistically significant differences between the two forms of biostatistics teaching. This applies to students in all fields of study. The severity of exam and student conduct stress for the traditional form was found to be statistically significantly higher (*p* < 0.001) compared to the practical method. According to the students of the study fields surveyed, the practical method of teaching biostatistics allows them to acquire practical knowledge to a greater extent (*p* < 0.001) and to be more satisfied after passing the exam in this subject. Respondents also stated that there may be difficulties in interpreting published research results to a greater extent (*p* < 0.001) with the traditional teaching method compared to the practical method.

**Table 2:** Descriptive statistics on the parameters analysed for the two forms of biostatistics teaching, i.e. traditional (1) and practical (2)

Variable		Study subject													
		Medical analytics		Dietetics		Pharmacy		Physiotherapy		Midwifery		Public health		Emergency medicine	
		1	2	1	2	1	2	1	2	1	2	1	2	1	2
Intensification of stress	M	68.31	27.04	65.92	24.8	68.61	27.05	69.75	29.28	70.82	30.32	72.65	32.63	73.24	30.33
	SD	16.59	10.44	18.93	14.94	18.27	12.76	20.93	12.18	18.7	14.42	20.06	14.12	18.56	13.39
	Me	70	29.5	65	20	70	25	73	27	70	30	75	30	80	25
	Q1	55	20	50.25	15	55	20	55	20	60	20	55	20	55	20
	Q3	80	35.25	80	34.25	80	35	81	40	87.25	35	90	43	85.75	40
Exertion of practical knowledge	M	38.29	75.14	40.56	71	41.5	73.45	43.14	77.91	37.98	70.55	38.46	73.49	41.59	77.14
	SD	17.5	17.35	20.31	21.1	20.65	21.7	19.75	18.27	37.5	70	16.98	19	19.83	19.8
	Me	35	80	45	70	40	80	45	80	20.18	21.71	40	70	40	85.5
	Q1	25	58.75	25	54.25	25	55	25	67.5	21.25	55	25	55	25.75	55
	Q3	46.25	90	55	90	55	90	55	90	48.75	90	55	90	55	90
Examination stress	M	66.54	22.99	65.71	22.62	62.91	22.34	67.45	27.28	66.36	17.98	64.71	18.18	68.66	24.28
	SD	22.35	19.12	21.49	15.9	22.99	16	23.05	18.43	22.09	16.57	25.25	14.03	21.78	15.86
	Me	70	20	60	20	60	20	70	20	67.5	15	55	15	70	20
	Q1	50	10	50	10	45	10	45	12.5	45	5	45	5	53.75	10
	Q3	85.75	30	87.75	35	80	30	90	40	88.75	20	90	20	90	40
Making it more difficult to interpret published research results in the future	M	62.11	17.63	61.76	19.56	64.71	21.49	61.62	19.45	60.21	17.48	67.97	24.96	68.83	20.88
	SD	22.36	9.92	23.7	12.88	24.15	15.79	22.74	15.81	24.39	13.76	23.99	15.03	23.16	15.07
	Me	60	20	55	20	61	20	55	20	55	10	60	20	70	20
	Q1	45	10	45	10	45	10	45	9.5	41.25	10	45	10	48.75	10
	Q3	80	20	90	23	90	25	80	20	83.75	23.75	90	40	90	26.25
Degree of satisfaction after passing the exam	M	41.21	80.81	37.06	76.18	36.19	81.23	33.83	84.16	30.25	75.71	33.59	75.38	32.59	74.26
	SD	15.82	14.34	18.8	18.4	17.4	16.75	18.4	14.71	15.37	18.72	16.25	18.63	15.57	19.22
	Me	40	80	35	80	35	90	35	90	30	75	35	80	30	75
	Q1	30	70	20	60	24	70	20	75	20	60	25	65	20	58.75
	Q3	50	90	45	90	50	90	45	95	40	90	45	90	45	90

M - mean, SD - standard deviation, Me - median, Q1 - first quartile, Q3 - third quartile

**Fig. 1:**
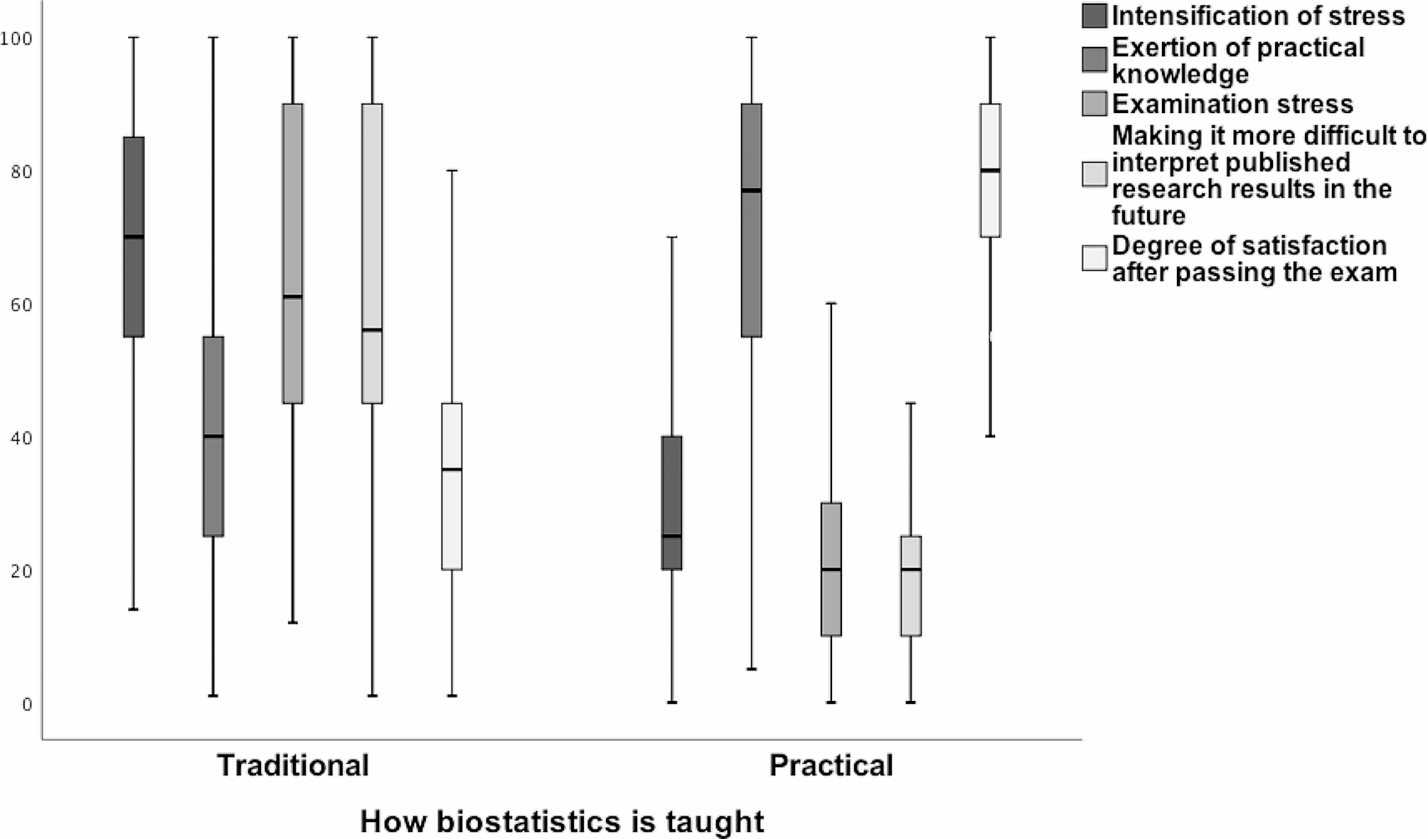
Analysed parameters for two forms of biostatistics teaching in the full group of students surveyed

Analysis with the Wilcoxon test showed that, across the entire group of students (Fig. 1), the severity of stress associated with teaching, taking an exam appeared to be statistically significantly lower for the practical teaching form compared to the traditional form (*p* < 0.001). The practical knowledge elevation and the degree of satisfaction after passing the exam were also found to be statistically significantly higher for the practical form (*p* < 0.001). In contrast, greater difficulty related to the future interpretation of published research results is characteristic of the traditional form (*p* < 0.001).

### Student opinion on possible ways to improve biostatistics education

Table 3 shows the opinion of the students in the different fields of study to the analysed parameters related to biostatistical quality improvement. A statistically significantly higher proportion of students in each field of study (*p* < 0.001) stated that medical school departments should require university staff to attend training in biostatistics. A greater proportion of them (*p* < 0.001) also believed that receiving training in biostatistics during their master’s/doctoral studies was sufficient to conduct statistical analyses independently in their future scientific work. The same applies to passing the course by carrying out a survey and then applying the statistical tests discussed in class and presenting the results to the course tutor. Also, a higher proportion of respondents (*p* < 0.001) stated that course credit could consist of a statistical review of the article provided by the subject instructor to assess the correctness of the analysis performed by the authors. Also of note is the fact indicating that a significantly higher proportion of students in each course confirmed that it should be possible to take an optional class in biostatistics (*p* < 0.001), to hear a lecture from an expert in conducting statistical analyses (*p* < 0.001) and that the classes taught should be based on the field of study being taken (*p* < 0.001). There was no statistically significant relationship between the field of study and the parameters shown in Table 2 (*p* > 0.05). In other words, a similar percentage of students from the different medical faculties supported each statement (Table 3).

**Table 3:** Student opinion on analysed variables for biostatistical quality improvement

Study subject	Medical analytics		Dietetics		Pharmacy		Physiotherapy		Midwifery		Public health		Emergency medicine	
	n	%	n	%	n	%	n	%	n	%	n	%	n	%
Medical school faculties should require university staff to attend training in biostatistics	67	96	78	93	105	95	65	94	51	91	75	95	55	95
Receiving training in biostatistics during master’s/doctoral studies is sufficient to conduct statistical analyses independently in future research work	1	1	2	2	6	5	4	6	2	4	6	8	5	9
The credit for the course could consist in conducting a survey on one’s own, applying con. a credit for the course could consist in conducting a survey on one’s own, applying several statistical tests presented during the classes and presenting the obtained results to the teacher and other students	65	93	75	89	95	86	58	84	52	93	69	87	50	86
A credit for the course could consist in reviewing published articles from the point of view of statistical correctness	50	71	67	80	83	75	57	83	45	80	69	88	43	74
Additional optional classes in biostatistics could be offered to willing students	67	96	83	99	109	98	68	99	55	98	78	99	57	98
Online lectures should be offered to students/scientists, during which experts would discuss the most important issues related to conducting statistical analysis	69	99	82	98	107	96	68	99	56	100	78	99	57	98
Practical conducting of classes in biostatistics should be based on the field of studies taken	69	99	82	98	110	99	67	97	56	100	78	99	58	100

**Fig. 2:**
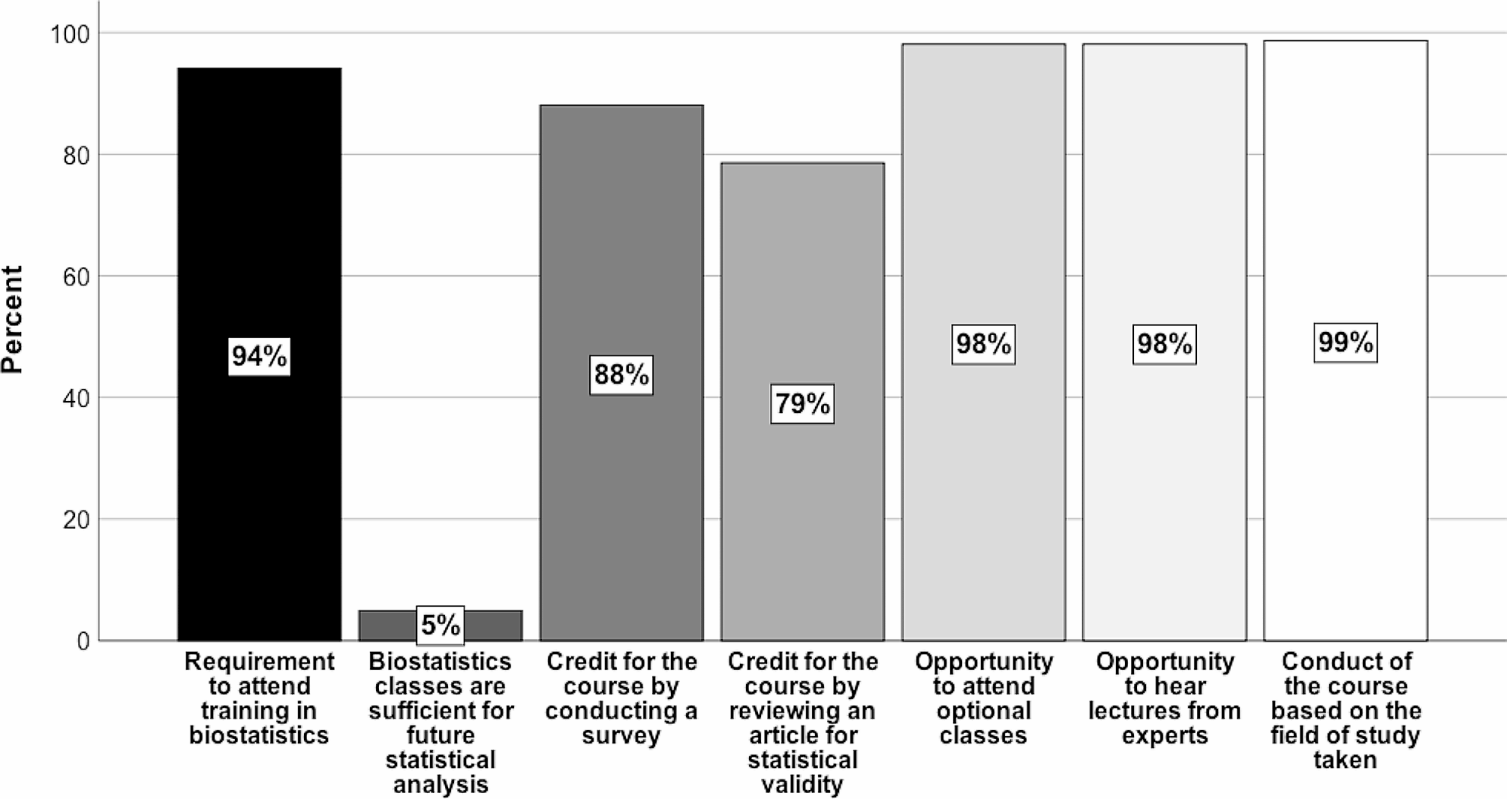
Opinion of all student groups on the parameters analysed related to biostatistical quality improvement

The chart below shows the percentage of support for the statements analysed, but across the entire student group. Among the 527 students, a statistically significantly higher proportion of them (*p* < 0.001) express the same opinion towards the individual statements as was the case for the individual fields of study (Fig. 2).

What is noteworthy is the statistically significant relationship between students’ opinion that biostatistics classes are sufficient for future statistical analysis and their opinion indicating that there should be a requirement among university employees to attend biostatistics training (*p* = 0.04). Among the students who believe that there is such a requirement, 95.6% also say that the biostatistics classes taught at university are not sufficient for future statistical analyses. Among students who believe that it should be possible to attend optional classes in biostatistics, 98.8% of them (*p* < 0.001) also believe that it should also be possible to listen to lectures given by experts in this field.

## Discussion

### Importance of practical biostatistics teaching

The survey carried out is the first on students’ opinions on the practical importance of the way statistics education is delivered at medical universities. The research published so far has focused on statistical recommendations that should be implemented to improve the quality of education in this area. This holds especially true for the recommendations outlined in the 2006 publication of PLOS BIOLOGY, where the authors aimed to pinpoint opportunities for enhancing the teaching of biostatistics in basic science [6]. Based on such recommendations, as well as the manuscript’s author’s many years of experience, a survey was conducted among a group of students from seven medical faculties in Poland, comparing the traditional form of teaching biostatistics with a practical one. The theoretical form consisted of carrying out a statistical analysis in the package based on a previously heard presentation, while the practical form consisted of students reviewing the article, indicating why the authors used the particular statistical test discussed in class. A further part of the practical form consisted of carrying out an analysis in the analysis package on the basis of the instruction in front of you, in which the step-by-step stages of the work were outlined.

The results indicated the validity of implementing a more practical method of teaching biostatistics in the future. For the practical form of teaching biostatistics, according to the students, less exam stress associated with teaching would be characteristic, as well as more satisfaction associated with it. In 2008, a survey of medical trainees indicated that up to 86% of them were dissatisfied with the biostatistics workshops conducted. During the didactic lectures, detailed presentations of various statistical concepts are provided, but insufficient attention is given to addressing specific research problems. Consequently, students engaged in such activities tend to retain minimal information and struggle to connect the acquired knowledge to their respective disciplines of study [18]. In a survey conducted in the US, only 17% said that the teaching of biostatistics was adequate [19]. As reported in the Journal of the American Medical Association, the survey results revealed that 75% of respondents expressed a lack of confidence in their statistical knowledge, feeling ill-equipped to meet the criteria for inclusion in medical journals [20]. The results obtained in this study showed that students are more able to acquire practical knowledge by participating in practical biostatistics classes. A survey of 138 s-year medical students revealed that a substantial 78.1% attributed the waning interest in biostatistics to a deficiency in practical teaching of the subject [21]. For this reason, it is recommended that emphasis be placed on more practical teaching of biostatistics, including reading articles in which the statistical test discussed in class has been applied. A larger proportion of students stated that additional training in biostatistics for researchers is necessary and that the traditional form of teaching may contribute to future difficulties in interpreting published research results. The same applies to the sentence indicating that college classes are not sufficient for independent statistical analysis in the future. The findings presented in MedEdPORTAL in 2021 suggest that participation in seminars with specialized statistical training contributes to a boost in self-confidence among a group of scientists in this field. Respondents expressed that attending the seminar proved to be highly beneficial for their day-to-day academic endeavors [22]. Regular participation in this type of training could in future help to reduce the percentage of accepted articles in which the statistical analysis is incorrectly performed. This is further substantiated by the findings of a study published in 2021, which disclosed that among 708 lecturers from 102 schools, the percentage of correct answers to questions related to conducting statistical analysis was only 66.2%. Hence, the study suggests the need for additional training [23]. Students in the various fields of study also indicated a desire for opportunities to participate in additional optional activities and to hear expert lectures on statistical analysis in the broadest sense. This only confirms the validity of implementing the type of recommendations that PLOS BIOLOGY identified. The authors recommend conducting statistical workshops involving students and statisticians. Training should be provided to students with a view to future employment [24, 25]. The results also confirmed the validity of the recommendations indicated in PLOS BIOLOGY for teaching biostatistics based on the field of study [6]. Conducting statistical analyses based on a specific area of medicine can contribute to greater involvement and interest of students in that specific area. Credit for the course, according to the students of the individual courses, should be based, for example, on carrying out a specific study and then, on the basis of instruction, applying the relevant test, as well as reviewing a published article in which the authors applied a specific statistical test. The efficacy of hands-on teaching methods in the subject can be extrapolated to other medical domains. As per information published in BMC Medical Education, a study involving 181 medical students engaged in surgical skills training and practice revealed that employing teaching assistants to instruct complex practical skills proved effective in both short- and long-term retention [26]. A methodological review conducted in 2016 on fundamental practical skills in medical studies suggested that the mandatory incorporation of multimedia applications, along with relevant exercises, positively influences the acquisition of practical skills [27]. For this reason, more practical teaching of the subject of biostatistics should be implemented in as many medical schools as possible. The most important recommendations related to the teaching of biostatistics that are recommended for implementation at medical universities are extracted below.

### Limitations

A limitation of the present study is that it was conducted in survey form. In the future, it would be advisable to conduct such studies during biostatistics classes to more comprehensively reflect the actual teaching experiences gained by students. However, in order to increase the credibility of the survey’s completion by students of specific medical faculties in Poland, the dean of a specific faculty was contacted first, who, with permission, forwarded the contact to the head of year, who then forwarded the survey to the students of his or her year via a dedicated email. Otherwise, it would be difficult to make contact with students from different medical faculties in several cities in Poland at the same time. One limitation of our study lies in the formulation of the vignettes, which may influence participant responses and should be acknowledged. The article gathers student perceptions, which should be triangulated with teachers’ opinions. Additionally, for enhancing the overall research value in the future, it is recommended to employ mixed research methods. As a limitation of this study, it is worth considering whether conducting a factor analysis in the future could yield more detailed and comprehensive results, particularly in understanding the key factors influencing students’ opinions regarding two forms of biostatistics instruction. Such an analysis could contribute to better defining relevant aspects, thereby serving as a valuable complement to the findings presented here.

## Conclusions and educational recommendations

It is recommended that biostatistics classes be conducted on the basis of reviewing an article in which the authors have applied a particular statistical test. Students would answer questions related to why exactly in this article one and not another test was used. The same applies to checking that the authors have examined the necessary assumptions to apply the test. Before students started reading the article, the subject leader would introduce the students to the specific statistical test discussed in class.

The second recommendation is to carry out the test in the statistical package on the basis of the manual in front of you, in which a step-by-step scheme for carrying out a specific test would be presented. After answering the question of why the authors applied a particular statistical test in the published manuscript, students could, based on this instruction, perform an exercise to apply the test in question based on the assignment received from the subject instructor. Instead of focusing on memorising dozens of procedures performed and the associated stress, students would take specific tests in a relaxed manner.

The third recommendation is related to course credit. This could be done by conducting a survey yourself, and then applying at least some of the statistical tests discussed in biostatistics classes, and then presenting the results obtained in front of the subject instructor and the other students. Another form of carrying out a course credit could be to review an article received from the subject instructor in which the authors applied the statistical tests discussed in class. The student would have the opportunity to provide a review covering all aspects related to the statistical analysis carried out by the authors of this manuscript.

The last recommendation concerns the delivery of courses based on the discipline within which students are studying. For example, physiotherapy students could learn the purpose of applying specific tests from manuscripts in which the authors examine various aspects related to this field of medicine. The same applies to then carrying out statistical tests yourself based on the instruction you have. This type of recommendation could certainly have a positive impact on students’ biostatistical knowledge.

### Electronic supplementary material

Below is the link to the electronic supplementary material.

Supplementary Material 1

## Data Availability

The datasets used and analysed during the current study are available from the corresponding author on reasonable request.
