# Supplementary material for: Enhancing biostatistics education for medical students in Poland: factors influencing perception and educational recommendations
Source: BMC Med Educ. 2024 Apr 22;24:428. doi: 10.1186/s12909-024-05389-z (PMC11034022; doi:10.1186/s12909-024-05389-z)
Supplement: Supplementary file 1 — Supplementary Material 1 [file 12909_2024_5389_MOESM1_ESM.pdf]

Appendix 1 (Vignette). A survey of traditional and practical forms of teaching biostatistics

1. Sex:

a) Female

b) Male

2. Age (years):

3. Place of residence:

a) Village

b) City

4. Study subject:

The survey was divided into two parts at the outset. Both parts refer to the chosen test, i.e. the t-student test for dependent samples.

First part

Imagine that the topic of the class is the t-student test for dependent samples. You learned from the subject leader that we use this test when we want to compare the averages of two measurements, i.e. in the same group of people. For example, we want to test whether the use of drug A for a certain period of time lowers total cholesterol levels in a group of people with ischaemic heart disease. You start running the test in the statistical package. The presenter gives a step-by-step presentation on how to carry out an analysis using this test, including the assumptions necessary for its application. The majority of the class is devoted to performing the analysis, while at the end of the class the interpretation of the results obtained is carried out. Then, approximately 30 minutes before the end of the class, you are to carry out the same test yourself step by step, i.e. perform a new task, i.e. examine the assumptions, perform the analysis and interpret the results. You don't have access to all the slides on which the way the test was conducted was presented, you wrote down in your notebook what you managed to do based on the presentation given by the tutor at short notice. Now tick how (0-100%) you support the

following statements regarding the severity of stress, how satisfied you are with the activities and the other statements.

5. Increased stress associated with carrying out a number of procedures in the statistical package:

6. Conducting classes in this way can contribute to the acquisition of practical knowledge from them:

7. Increased stress related to subsequent classes conducted in this way, and consequently fear of the subject exam awaiting in the session:

8. Teaching in this way may make it more difficult in the future to interpret researchers' published findings:

9. Indicate your level of satisfaction with the subject after passing the exam:

Second part

Imagine that you are carrying out the same task as in point one, however, the activity is carried out in a completely different way. At the outset, the subject leader indicates what the objective is in relation to the use of the t-student test for dependent samples. He then provides each student with a publication in which the authors have applied this particular test. Below screenshots of such a publication together with the research results obtained were placed. You proceed to read it, analyse it. The authors wanted to investigate, in the same group of patients, the effectiveness of dietary treatment, whether it affects lipid parameters (front page, first part of screen). One of the tests they indicate is the dependent sample Student's t-test, discussed in class. It can be seen that the authors checked that there is a met assumption of normality of distribution. If it was met, they knew they could apply this particular test (statistical analyses, second part of screen). Knowing what the test is for, about the authors' examination of their assumptions, you move on to the results they present. You want to find out if there is a statistically significant change after 12 months of treatment. As it can be seen from the attached Table III, in terms of 3 of the 4 parameters, the p-value is lower than 0.05, so there has been a statistically significant change.

The results obtained after 12 months of treatment were found to be statistically significantly lower (results obtained, third part of screen).

Screen 1: Abstract of published article [28]

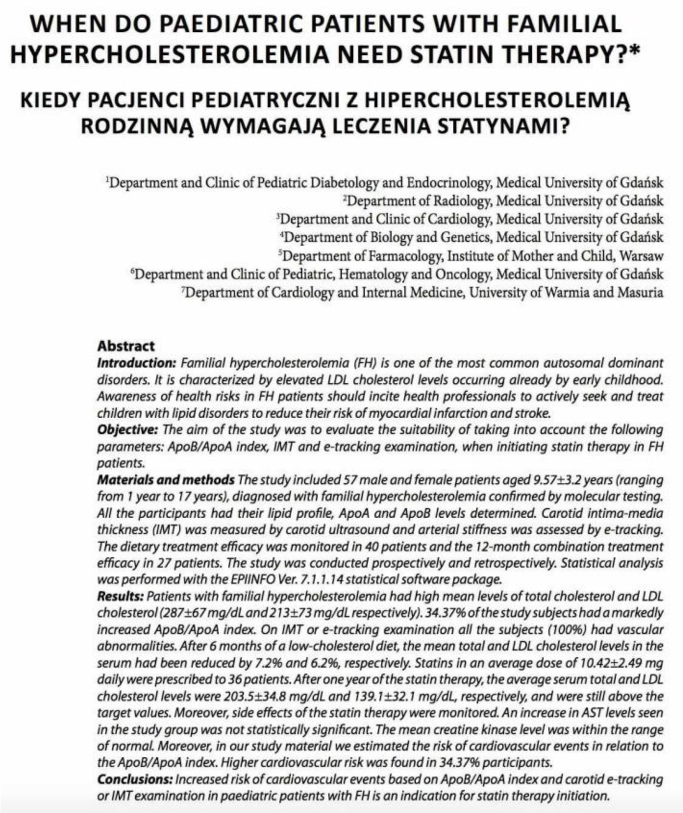

WHEN DO PAEDIATRIC PATIENTS WITH FAMILIAL HYPERCHOLESTEROLEMIA NEED STATIN THERAPY?*

KIEDY PACJENCI PEDIATRYCZNI Z HIPERCHOLESTEROLEMIĄ RODZINNĄ WYMAGAJĄ LECZENIA STATYNAMI?

[1]Department and Clinic of Pediatric Diabetology and Endocrinology, Medical University of Gdańsk
[2]Department of Radiology, Medical University of Gdańsk
[3]Department and Clinic of Cardiology, Medical University of Gdańsk
[4]Department of Biology and Genetics, Medical University of Gdańsk
[5]Department of Farmacology, Institute of Mother and Child, Warsaw
[6]Department and Clinic of Pediatric, Hematology and Oncology, Medical University of Gdańsk
[7]Department of Cardiology and Internal Medicine, University of Warmia and Masuria

Abstract
Introduction: *Familial hypercholesterolemia (FH) is one of the most common autosomal dominant disorders. It is characterized by elevated LDL cholesterol levels occurring already by early childhood. Awareness of health risks in FH patients should incite health professionals to actively seek and treat children with lipid disorders to reduce their risk of myocardial infarction and stroke.*
Objective: *The aim of the study was to evaluate the suitability of taking into account the following parameters: ApoB/ApoA index, IMT and e-tracking examination, when initiating statin therapy in FH patients.*
Materials and methods *The study included 57 male and female patients aged 9.57±3.2 years (ranging from 1 year to 17 years), diagnosed with familial hypercholesterolemia confirmed by molecular testing. All the participants had their lipid profile, ApoA and ApoB levels determined. Carotid intima-media thickness (IMT) was measured by carotid ultrasound and arterial stiffness was assessed by e-tracking. The dietary treatment efficacy was monitored in 40 patients and the 12-month combination treatment efficacy in 27 patients. The study was conducted prospectively and retrospectively. Statistical analysis was performed with the EPIINFO Ver. 7.1.1.14 statistical software package.*
Results: *Patients with familial hypercholesterolemia had high mean levels of total cholesterol and LDL cholesterol (287±67 mg/dL and 213±73 mg/dL respectively). 34.37% of the study subjects had a markedly increased ApoB/ApoA index. On IMT or e-tracking examination all the subjects (100%) had vascular abnormalities. After 6 months of a low-cholesterol diet, the mean total and LDL cholesterol levels in the serum had been reduced by 7.2% and 6.2%, respectively. Statins in an average dose of 10.42±2.49 mg daily were prescribed to 36 patients. After one year of the statin therapy, the average serum total and LDL cholesterol levels were 203.5±34.8 mg/dL and 139.1±32.1 mg/dL, respectively, and were still above the target values. Moreover, side effects of the statin therapy were monitored. An increase in AST levels seen in the study group was not statistically significant. The mean creatine kinase level was within the range of normal. Moreover, in our study material we estimated the risk of cardiovascular events in relation to the ApoB/ApoA index. Higher cardiovascular risk was found in 34.37% participants.*
Conclusions: *Increased risk of cardiovascular events based on ApoB/ApoA index and carotid e-tracking or IMT examination in paediatric patients with FH is an indication for statin therapy initiation.*

Screen 2: Description of the statistical tests applied, including the t-student test for dependent samples

Statistical analysis

The findings of the study were analysed by statistical methods to verify our hypothesis. The hypothesis on the equality of means from all samples was verified by ANOVA or by the nonparametric Kruskal-Wallis sum-rank test (for anomalous groups or groups of small number of cases); the homogeneity of variance was estimated by Bartlett's test.

The hypothesis that the parameter means in dependent samples (before and after the treatment) are equal was verified by Student's t-test for paired samples for groups with normal distribution, and by Wilcoxon signed-rank test when populations cannot be assumed to be normally distributed; normality was tested by the Shapiro-Wilk test.

For specified pairs of parameters the correlation analysis was done by calculating the Pearson correlation coefficient r, where P ‹ 0.05 was considered statistically significant. Statistical analysis was performed using a statistical program package EPIINFO Ver. 7.1.1.14 (of 2nd July 2013).

Screen 3: Results obtained

Table III. Changes in lipid levels during their (12 month) combination therapy with diet and statins n=27.
Tabela III. Zmiany stężenia lipidów w trakcie leczenia dietą i statynami w ciągu 12 miesięcy.

	**Total Cholesterol** ***Cholesterol całkowity mg/dl***	**LDL mg/dl**	**HDL mg/dl**	**TG mg/dl**
Diet<br>Dieta	289.4±59.84	212.2±71.16	58.22±31.9	101.2±64.3
12 month statin treatment<br>*12 miesięcy leczenia statnami*	203.5±34.8	139.1±32.1	50.55±9.65	72.6±33.37
p	0.0000006	0.0000006	0.455903	0.001453

When you have finished analysing the publication, you answer the subject leader why the authors in the publication applied such a test, whether its assumptions were met and what the results indicate.

Now you are given an assignment and in the statistical package yourself you are supposed to check whether, for example, in a group of people with depression, participation in psychotherapy improves their quality of life as measured by the Beck Depression Scale. You have a printed manual in front of you, which you can also have in future colloquia and examinations. The purpose of this instruction during the examination would be to carry out a statistical test selected on the basis of your knowledge necessary for the task obtained. This

step-by-step guide shows how to carry out a specific test in the package and examines the assumptions necessary for its application. Below you have this type of sample screenshot made available. You don't have to focus on writing down individual procedures in a notebook.

You start testing the assumption of normality of distribution, click on analysis, then statistical description and exploration.

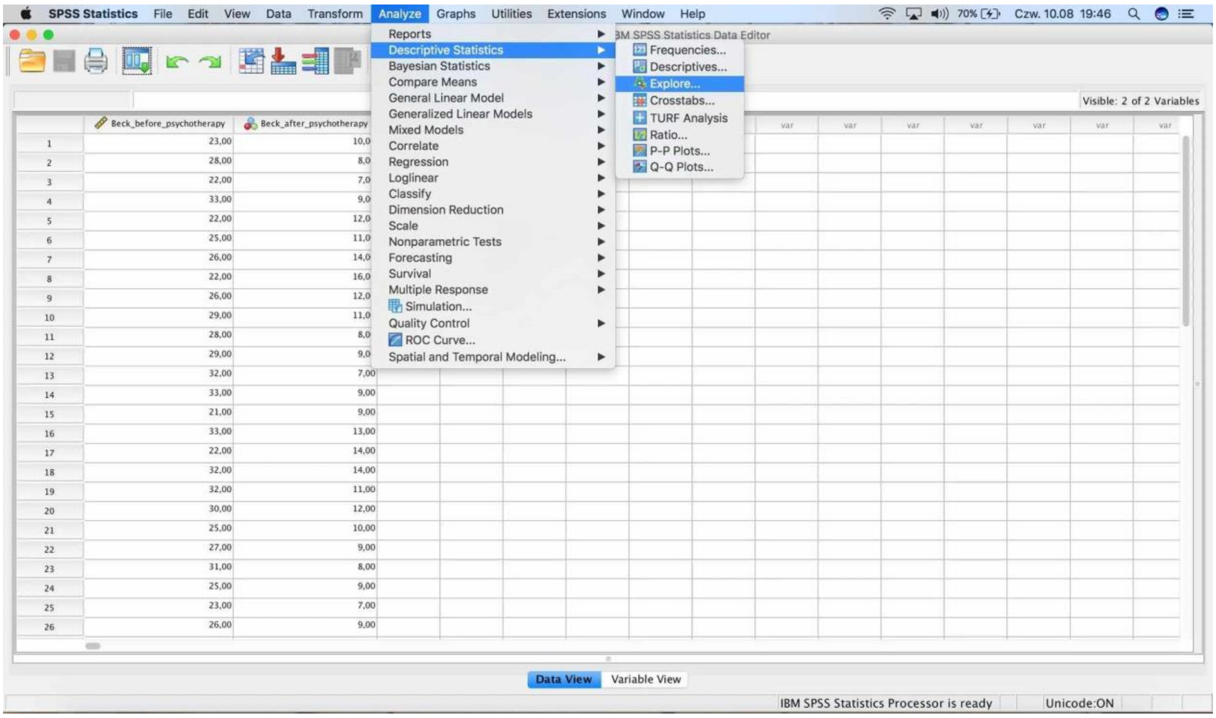

In the dependent variables field you flip the Beck Depression Scale scores before and after therapy from the left. You then click on plots.

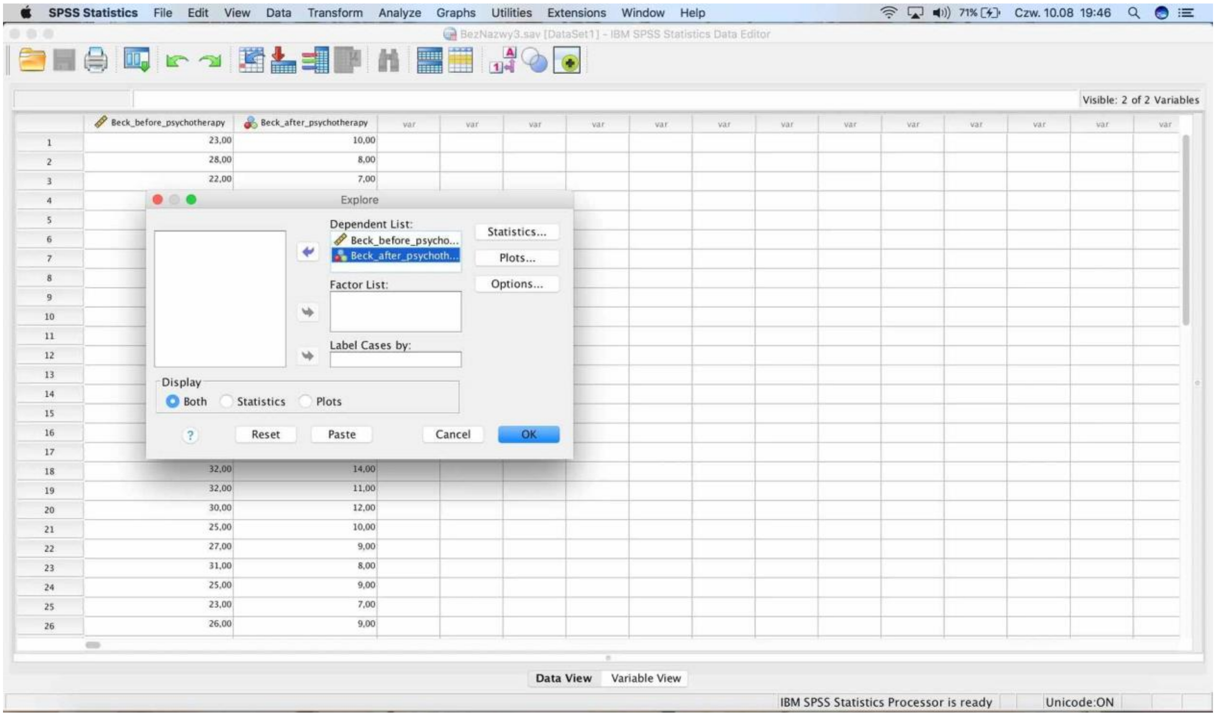

You click on the graphs of the normality plots with tests and then continue.

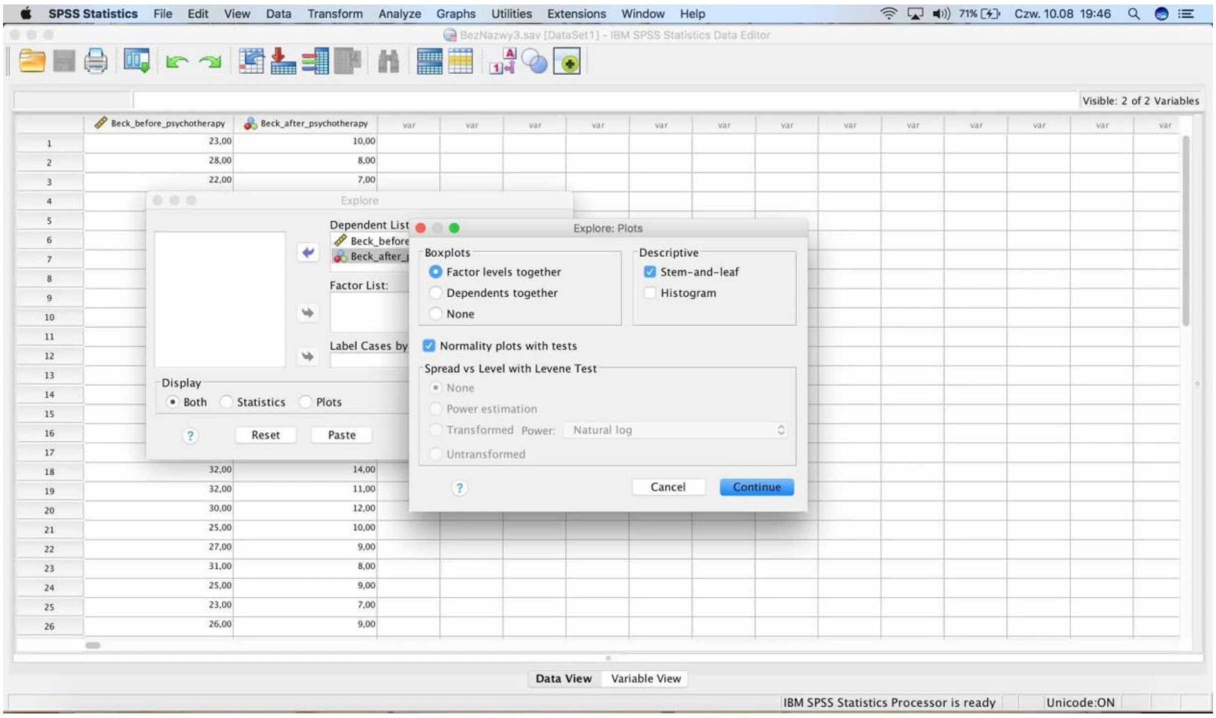

You click ok.

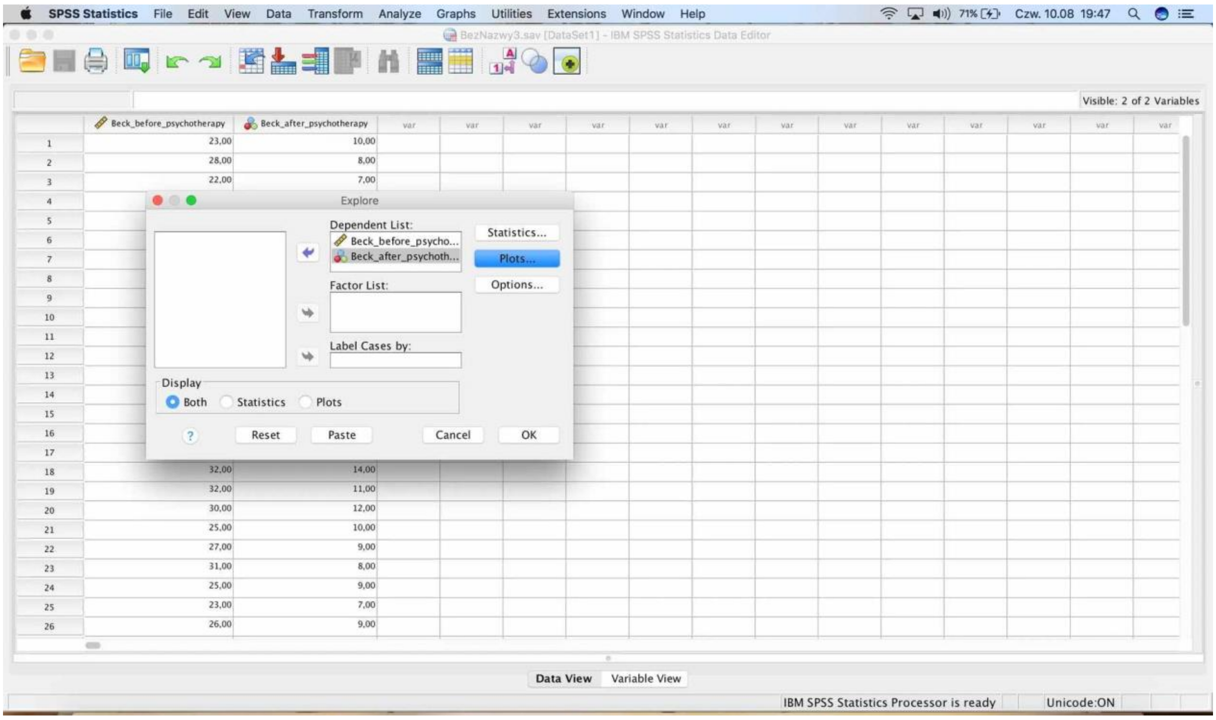

You see that the significance for the Shapiro-Wilk test is greater than 0.05, and you have learned from the instructor and according to the information posted in the manual that this means that the assumption of normality of the distribution is then satisfied.

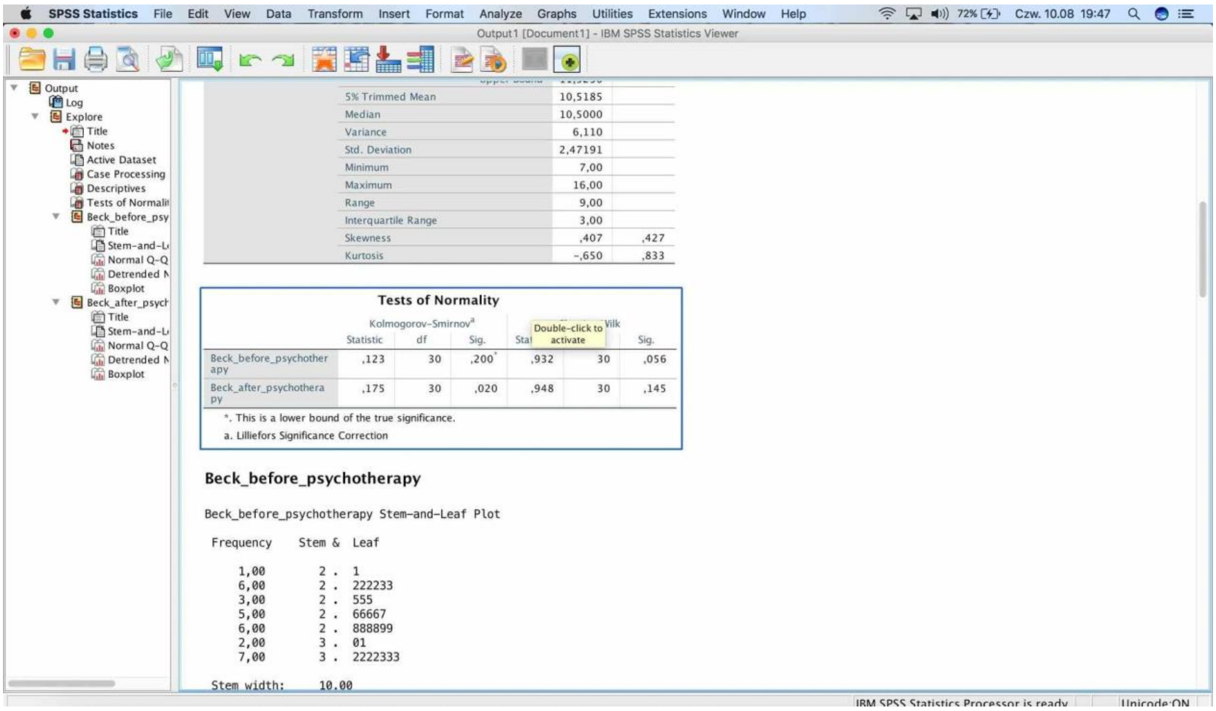

Now that you know that the assumption is met, you proceed to carry out the test. You click analysis, t-test for dependent samples.

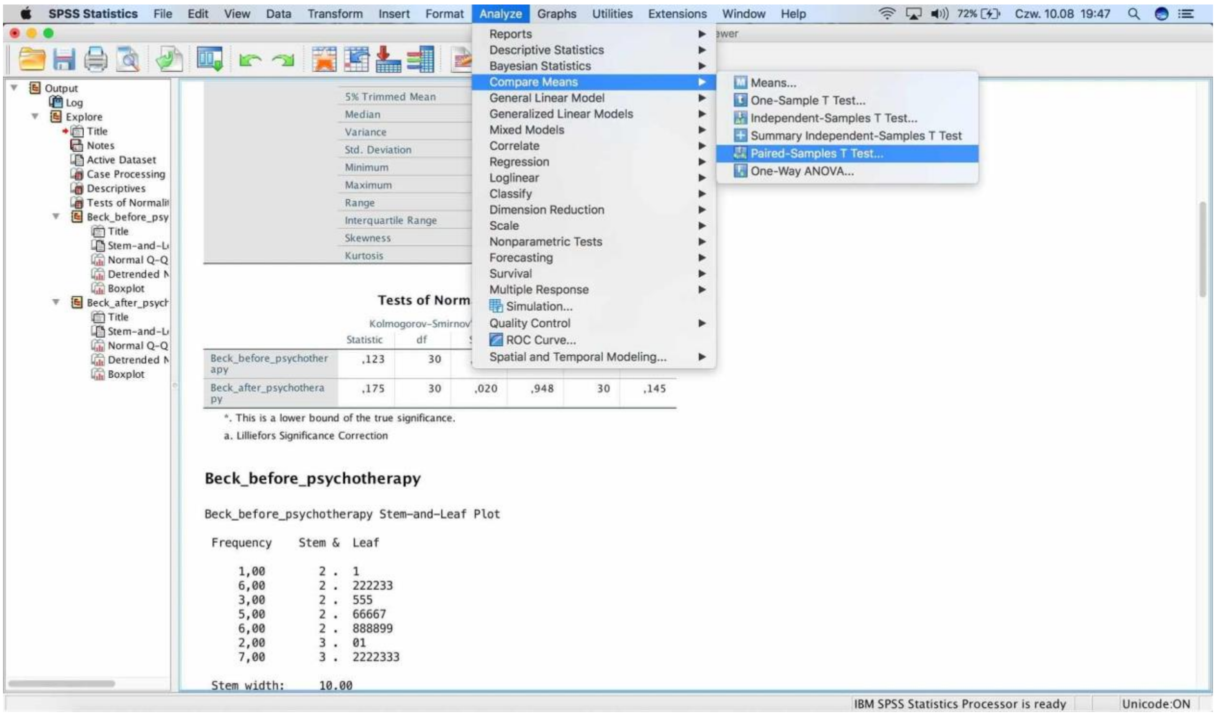

You transfer to the pair of variables first the first measure of the Beck Depression Scale.

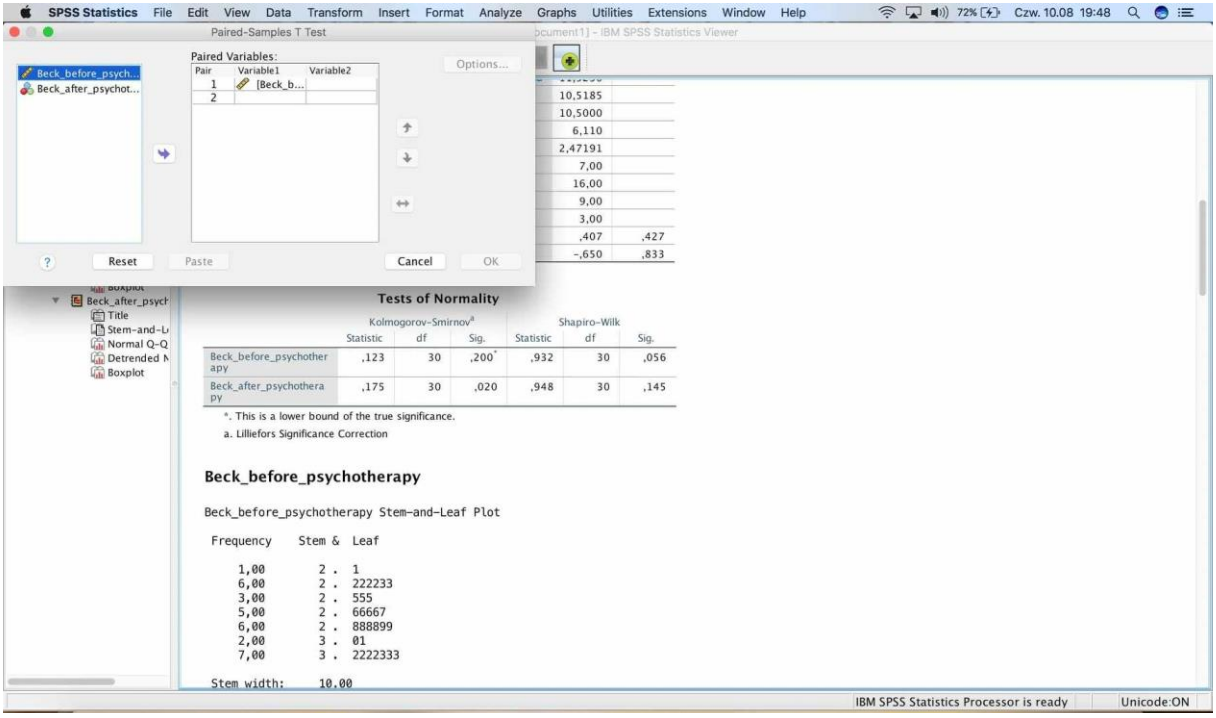

You then flip the variable denoting the results for the second measurement, i.e. after the psychotherapy applied, and click ok.

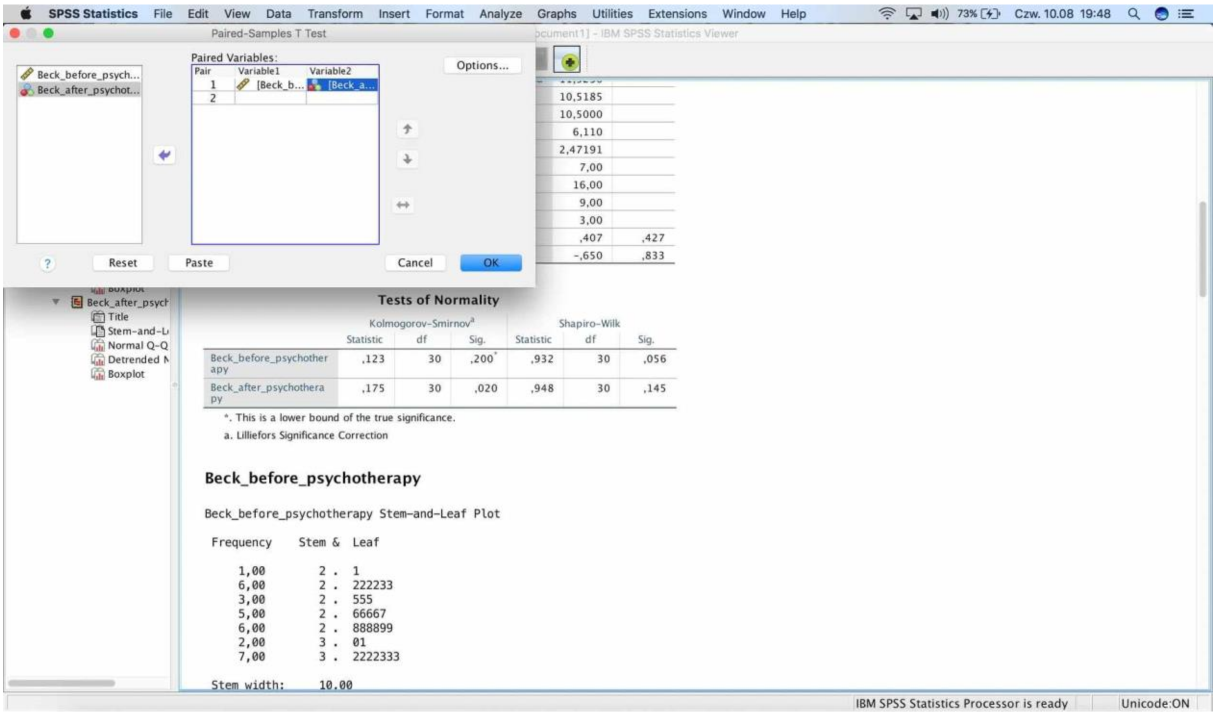

You begin to interpret the test results obtained. You can see that the p is less than 0.05, and therefore, based on the words of the subject leader and the instruction in front of you, this means that there are statistically significant differences, so the Beck depression scale scores after the psychotherapy applied turned out to be statistically significantly lower.

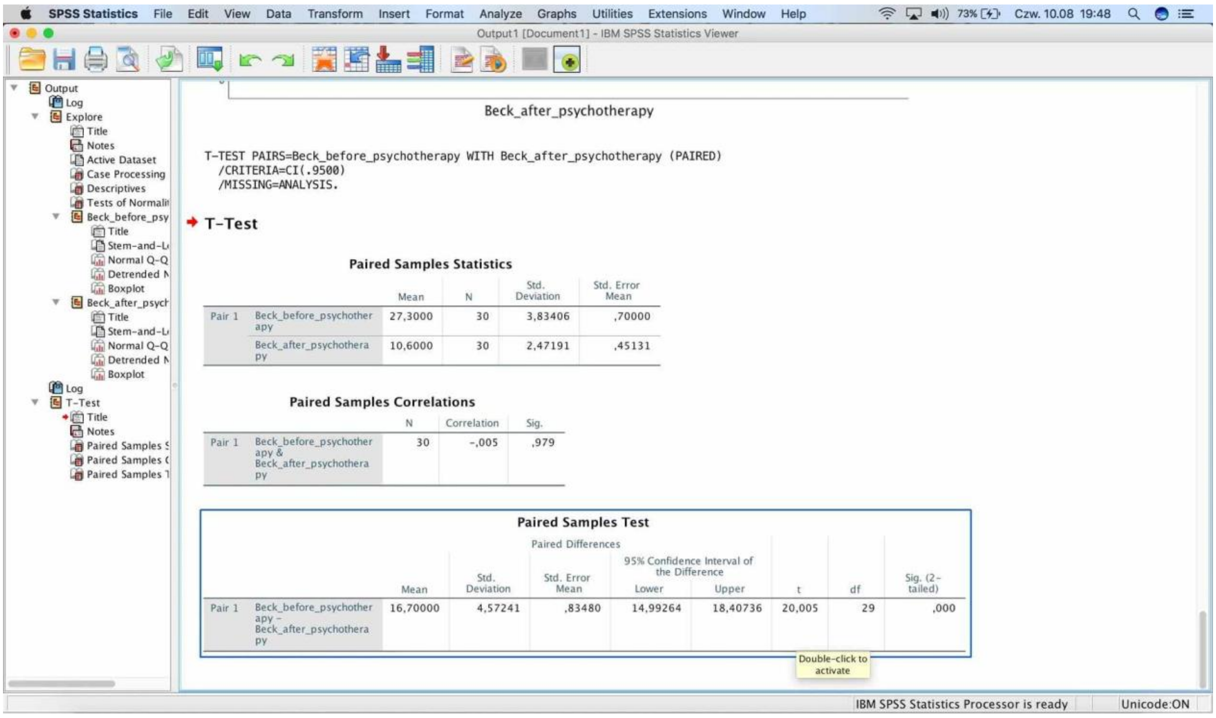

You record the results obtained, for example, as follows:

The mean Beck Depression Scale score after psychotherapy in the study group was found to be statistically significantly lower (the level of statistical significance is less than 0.001). You know from the manual in front of you that such an entry is made when the p-value is 0.

You now look at the descriptive statistics and see that in the second time period the mean obtained was statistically significantly lower (M = 10.6; SD = 2.47) compared to the period before psychotherapy started (M = 27.3; SD = 3.83).

Now answer the same questions as above.

10. Increased stress associated with carrying out a number of procedures in the statistical package (with instruction):

11. Conducting classes in this way can contribute to taking practical knowledge out of them:

12. Increased stress related to the next class conducted in this way, and consequently the fear of the subject exam awaiting in the session:

13. Teaching in this way may make it more difficult in the future to interpret the researchers' published findings:

14. Indicate your level of satisfaction with the subject after passing the exam:

The last part of the survey includes some additional questions

15. Do you think university medical faculties should require staff to attend statistics training:

a) Yes

b) No

16. Do you think that receiving training in statistics during your master's/doctoral studies is sufficient to carry out statistical analyses independently in your future academic work:

a) Yes

b) No

17. In your opinion, could a credit for the course consist in carrying out a survey (e.g. a questionnaire) on your own, applying con. several statistical tests presented during the course and presenting the obtained results to the instructor and other students? An exemplary outcome of such work could be a publication in which the student co-authored:

a) Yes

b) No

18. In your opinion, could a course credit consist of reviewing published articles for statistical validity? In other words, your role would be to assess the validity of the statistical tests used by the authors (and their assumptions) in the sample publications provided by the presenter for you:

a) Yes

b) No

19. In your opinion, could additional optional classes in statistics be held for willing students:

a) Yes

b) No

20. In your opinion, should online lectures be offered for students/scientists where statisticians would discuss the most important issues in conducting statistical analysis:

a) Yes

b) No

21. In your opinion, should the practical delivery of statistics classes be based on your field of study:

a) Yes

b) No
